# Supplementary material for: Identification of photoperiod-induced specific miRNAs in the adrenal glands of Sunite sheep (Ovis aries)
Source: Front Vet Sci. 2022 Jul 22;9:888207. doi: 10.3389/fvets.2022.888207 (PMC9354845; doi:10.3389/fvets.2022.888207)
Supplement: Supplementary file 1 [file Data_Sheet_1.ZIP › Supplementary file/miRDeep2 file.zip!/miRDeep2 file/Know_Structure/LP42A3/oar-mir-376a.pdf]

miRBase precursor : oar-mir-376a
Total read count : 343
oar-miR-376a-5p read count : 227
oar-miR-376a-3p read count : 113
remaining reads : 3

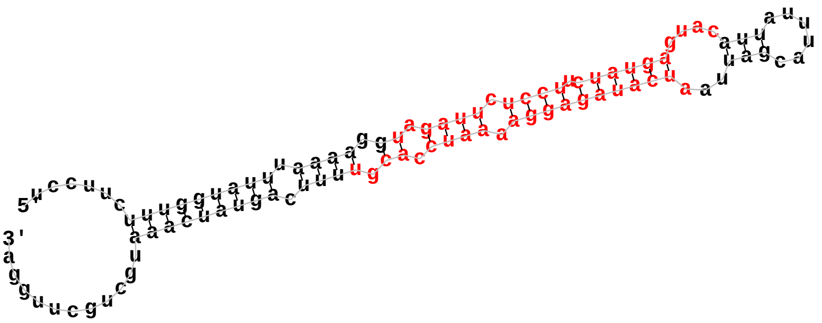

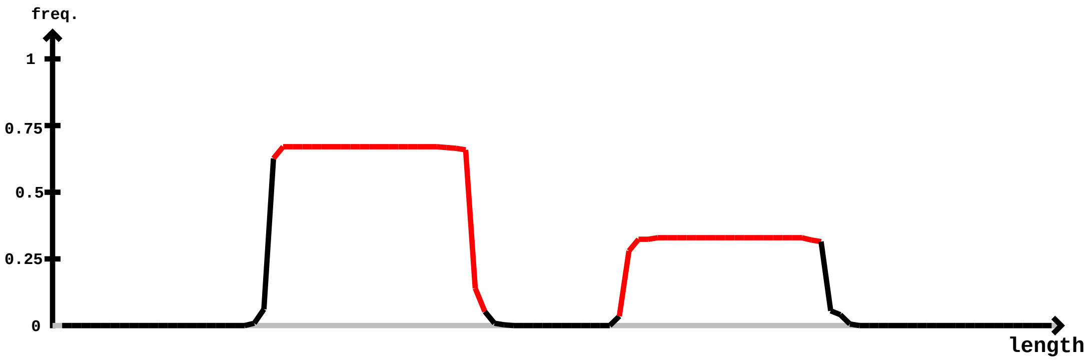

oar-miR-376a-5p

oar-miR-376a-3p

```
5'- uccuucuuugguauuuaaaagguagauucuccuucuaugaguacauuauuuacgauuaaucauagaggaaaauccacguuuucaguaucaaaugcugcuugga   -3'    exp
    ......((((((((.((((.((.((((.((((.((((((....(((......)))...)))))))))).)))).)).)))).)))))))).............    reads   mm     sample
    ...................agguagauucuccuucuauga...............................................................    1       0      seq
    ...................agguagauucuccuucuaugagu.............................................................    2       0      seq
    ....................gguagauucuccuucuaugagu.............................................................    18      0      seq
    .....................guagauucuccuucuaug................................................................    1       0      seq
    .....................guagauucuccuucuaugag..............................................................    2       0      seq
    .....................guagauucuccuucuaugagu.............................................................    146     0      seq
    .....................guagauucuccuucuaugagua............................................................    28      0      seq
    .....................guagauucuccuucuaugaguac...........................................................    15      0      seq
    .....................guagauucuccuucuaugaguaca..........................................................    2       0      seq
    ......................uagauucuccuucuaugagu.............................................................    12      0      seq
    ......................uagauucuccuucuaugagua............................................................    2       0      seq
    ......................uagauucuccuucuaugaguacau.........................................................    1       0      seq
    .........................................................aaucauagaggaaaauccacg.........................    1       0      seq
    .........................................................aaucauagaggaaaauccacgu........................    11      0      seq
    ..........................................................aucauagaggaaaauccac..........................    3       0      seq
    ..........................................................aucauagaggaaaauccacg.........................    1       0      seq
    ..........................................................aucauagaggaaaauccacgu........................    77      0      seq
    ..........................................................aucauagaggaaaauccacguu.......................    3       0      seq
    ...........................................................ucauagaggaaaauccacgu........................    1       0      seq
    ...........................................................ucauagaggaaaauccacguu.......................    2       0      seq
    ...........................................................ucauagaggaaaauccacguuu......................    11      0      seq
    ...........................................................ucauagaggaaaauccacguuuu.....................    1       0      seq
    .............................................................auagaggaaaauccacguuu......................    1       0      seq
    .............................................................auagaggaaaauccacguuuu.....................    1       0      seq
```
